# Correlation of Postoperative Shoulder Tip Pain in Low- Versus Standard-Pressure Pneumoperitoneum During Laparoscopic Cholecystectomy

**DOI:** 10.7759/cureus.114736

**Published:** 2026-08-18

**Authors:** Rakshita Saini, Mohit K Badgurjar, Mitesh Asari, Suman Parihar, Jay P Patel, Isha Purohit

**Affiliations:** 1 Department of General Surgery, Geetanjali Medical College and Hospital, Udaipur, IND

**Keywords:** cholelithiasis, co2 pneumoperitoneum, laparoscopic cholecystectomy, low-pressure pneumoperitoneum, postoperative shoulder tip pain, standard-pressure pneumoperitoneum

## Abstract

Introduction

During laparoscopic cholecystectomy, pneumoperitoneum is created to facilitate exposure of the operative site. It provides the surgeon with adequate visualization; however, it has been associated with postoperative shoulder tip pain. As an alternative, low-pressure pneumoperitoneum (8-10 mmHg) has been proposed. This study compared the postoperative shoulder tip pain between standard- and low-pressure pneumoperitoneum created during laparoscopic cholecystectomy.

Methods

This was a prospective comparative study conducted in the Department of General Surgery over 18 months, following approval from the institutional ethics committee. After sample size calculation, 80 patients were enrolled who underwent elective laparoscopic cholecystectomy for uncomplicated cholelithiasis. They were randomly allocated into two equal groups: Group A (standard-pressure pneumoperitoneum) and Group B (low-pressure pneumoperitoneum). Postoperative shoulder tip pain and abdominal pain were assessed using the Visual Analog Scale. Additionally, demographic variables, operative time, intraoperative complications, and hospital stay duration were recorded.

Results

The mean intra-abdominal pressure (IAP) was significantly higher in Group A (11.51 ± 0.28 mmHg) than in Group B (9.50 ± 0.27 mmHg, p = 0.001). There were no reported instances of postoperative shoulder tip pain. Postoperative abdominal pain was recorded at 6, 12, and 24 hours, but it was comparable between both groups with no statistically significant differences. No statistically significant difference was observed for operative time, intraoperative complications, hospital stay, and postoperative complications between both groups.

Conclusions

No significant difference was noted between postoperative shoulder tip or abdominal pain in low- and standard-pressure pneumoperitoneum in patients undergoing laparoscopic cholecystectomy. Our findings suggest that pressure selection in laparoscopic cholecystectomy may be guided by surgeon preference rather than by differences in postoperative pain.

## Introduction

Laparoscopic surgery, also called minimally invasive surgery, is a modern surgical technique that offers reduced postoperative pain, minimal blood loss, smaller scars, faster recovery, and a lower risk of infection compared to open surgery [1]. To facilitate such surgeries, there is a need to create pneumoperitoneum. 

Pneumoperitoneum is the controlled insufflation of gas, typically carbon dioxide (CO_2_), into the peritoneal cavity to create an adequate surgical operative field [2]. To ensure a stable operative environment throughout the surgery, a specialized insufflator maintains a stable intra-abdominal pressure (IAP) between 12 and 14 mmHg [3].

However, pneumoperitoneum has been associated with complications. These include compromised lung compliance and venous return due to diaphragmatic elevation, organ or vascular injury during the initial blind insertion phase, and the possibility of referred shoulder pain postoperatively due to residual diaphragmatic irritation from the absorbed CO_2_ [4]. Amongst these, postoperative shoulder tip pain is a particularly frequent complaint, which has been associated with high IAP [5]. Therefore, as an alternative, low-pressure pneumoperitoneum (8-10 mmHg) has been proposed to reduce postoperative pain while maintaining satisfactory surgical conditions.

Cholelithiasis is one of the most common gastrointestinal diseases, affecting approximately 6% of the global population [6]. In such cases, laparoscopic cholecystectomy remains the definitive and preferred treatment of choice [7]. For this procedure, the standard-pressure pneumoperitoneum is considered to be around 12 to 14 mmHg [8]. Because laparoscopic cholecystectomy is a standardized and routinely performed procedure, it allows an unbiased comparison between the two pneumoperitoneum pressures.

Although several studies have suggested that low-pressure pneumoperitoneum reduces postoperative pain, concerns remain regarding adequate operative exposure and technical safety [8,9]. Further evidence is required to determine whether lower insufflation pressures improve patient outcomes without compromising surgical performance. Therefore, the primary outcome of the present study aimed to compare postoperative shoulder tip pain between low- and standard-pressure pneumoperitoneum during laparoscopic cholecystectomy. The secondary objective included assessment of postoperative abdominal pain, operative time, hospital stay, and intraoperative complications between groups.

## Materials and methods

Study setting 

This was a prospective comparative study that began after obtaining approval from the institute’s Human Research Ethics Committee (HREC) in adherence to the Declaration of Helsinki (approval no. GU/HREC/EC/2024/2463). The study was conducted in the Department of General Surgery at Geetanjali Medical College and Hospital, Udaipur, over a period of 18 months, from April 30, 2024, to October 30, 2025. 

Patient selection and eligibility criteria 

Patients were evaluated and selected for laparoscopic cholecystectomy from the General Surgery outpatient department. Those meeting the inclusion and exclusion criteria were admitted and randomly allocated to two groups using a computer-generated random number table prepared by the Principal Investigator. The allocation sequence was concealed using an opaque envelope, opened only after enrolment of the patient into the group. However, due to the nature of the study, the surgeon and anesthesiologist could not be blinded. The investigator who assessed the postoperative pain was blinded to group allocation. Group A consisted of the standard-pressure pneumoperitoneum cohort, and Group B consisted of the low-pressure pneumoperitoneum cohort. Written informed consent was obtained from the patients before participation. The eligibility criteria for patient selection are presented in Table 1.

**Table 1:** Inclusion and Exclusion Criteria for Patient Selection.

Inclusion criteria	Exclusion criteria
Age ≥18 years	Patients unfit for general anesthesia
Diagnosed with uncomplicated cholelithiasis	Complicated cholelithiasis (e.g., acute cholecystitis, choledocholithiasis, gallstone pancreatitis)
Undergoing elective simple laparoscopic cholecystectomy	Difficult laparoscopic cholecystectomy requiring deviation from the standard procedure or conversion to open surgery
Provided written informed consent	
Patients with normal laboratory parameters	

Calculation of mean IAP

Pneumoperitoneum was created using the closed (Veress needle) technique. A small incision was created at the umbilicus, and a spring-loaded Veress needle was blindly inserted into the abdominal cavity. Standard safety checks were performed to confirm the correct placement of the needle. After the confirmation of proper placement, a laparoscopic insufflator delivered CO₂ gas into the peritoneal cavity. In order to account for pressure differences due to instrument manipulation, smoke evacuation, and suction, the IAP was measured at 15-minute intervals and recorded. These values were then used to calculate the mean IAP.

Study design

A detailed history was obtained for each patient, and a thorough general physical and systemic examination was performed using a pre-designed proforma. Postoperative shoulder tip pain was assessed using the Visual Analog Scale (VAS) at 6, 12, and 24 hours. All patients in both study groups received a standardized postoperative analgesic protocol, which included 1 gm paracetamol administered intravenously three times daily, and 75 mg diclofenac administered intravenously in 100 mL of normal saline twice daily. On the second day, the patients were switched to oral medication, which included a fixed-dose combination of paracetamol (325 mg), diclofenac (50 mg), and serratiopeptidase (15 mg), given twice daily. Tramadol was reserved as rescue analgesia for patients with a VAS score of more than 7; however, no patient in either study group required it.

Sample size calculation

Sample size was calculated using the formula for comparing two independent proportions at a 95% confidence level (Zα/2 = 1.96) and 80% power (Zβ = 0.8413). Based on the incidence of postoperative shoulder tip pain of 7.14% in the low-pressure pneumoperitoneum group and 28.57% in the standard-pressure pneumoperitoneum group, the minimum sample size in each group was calculated to be 32 patients in each group [10]. However, 40 patients were enrolled in each group, making a total sample size of 80 patients.

Statistical analysis

Data were collected and analyzed using Microsoft® Excel for Mac, version 16.110.3 (26070318), licensed under Office Home 2024 (Microsoft Corporation; Redmond, WA, USA). Continuous variables are expressed as mean ± standard deviation and compared using the unpaired t-test. Categorical variables are expressed as frequencies and percentages and compared using the chi-square test or Fisher's exact test where expected cell counts were small. A p-value < 0.05 was considered statistically significant.

## Results

Patient characteristics and demographic variables

After the application of inclusion and exclusion criteria, a total of 80 patients were included in the statistical analysis, with an equal number of participants in Group A and Group B (n = 40, 50%). All baseline demographic characteristics, such as age and gender, were comparable between both groups with no statistical significance (p > 0.05). Additionally, comorbidities, such as diabetes mellitus, hypertension, hypothyroidism, and asthma, were similarly distributed between the two groups. The absence of a statistically significant difference between the two groups suggests that they were both comparable with respect to demographic characteristics, making it unlikely that these variables acted as a confounding factor in this study. The demographic characteristics are presented in Table 2.

**Table 2:** Demographic Characteristics of Study Participants in Group A and Group B. #Differences in age between Group A (standard-pressure pneumoperitoneum) and Group B (low-pressure pneumoperitoneum) were compared using the unpaired t-test, while differences in gender and comorbidities were analyzed using the chi-square (χ²) and Fisher's exact test. SD: standard deviation

Characteristic	Group A	Group B	Test statistic^#^	p-value
Age (mean ± SD)	40.12 ± 12.89	42.05 ± 7.48	t-test = 0.65	0.54
Male, n (%)	16 (40)	15 (37.5)	χ² value = 0.65	0.74
Female, n (%)	24 (60)	25 (62.5)		
Diabetes mellitus, n (%)	2 (5)	2 (5)	Fisher's exact	0.80
Hypertension, n (%)	9 (22.5)	9 (22.5)		
Diabetes mellitus and hypertension, n (%)	4 (10)	6 (15)		
Hypothyroidism, n (%)	1 (2.5)	1 (2.5)		
Asthma, n (%)	0 (0)	2 (5)		
None, n (%)	24 (60)	20 (50)		

Ultrasonography (USG) findings of the gallbladder (GB) and common bile duct (CBD)

The perioperative ultrasonography findings showed that most patients in both groups had normal gallbladder (GB) thickness and common bile duct (CBD) appearance. Minor abnormalities, such as contracted or edematous GB wall, borderline CBD prominence, and bowel gas obscuration, were seen in some patients. However, both p-values were more than 0.05, indicating no statistically significant difference between the two groups. The ultrasonography (USG) findings of the GB and CBD are presented in Table 3. 

**Table 3:** Ultrasonography (USG) Findings of Gallbladder (GB) and Common Bile Duct (CBD) in Group A and Group B. Differences in ultrasonography findings between Group A (standard-pressure pneumoperitoneum) and Group B (low-pressure pneumoperitoneum) were compared using Fisher's exact test.

USG parameter	USG finding	Group A, n (%)	Group B, n (%)	p-value
GB thickness	Contracted	1 (2.5)	1 (2.5)	0.68
	Edematous	1 (2.5)	2 (5.0)	
	Thickening	0 (0.0)	1 (2.5)	
	Normal	38 (95.0)	36 (90.0)	
CBD	Borderline prominent	1 (2.5)	1 (2.5)	0.62
	Obscured by bowel gas	2 (5.0)	0 (0.0)	
	Normal	37 (92.5)	39 (97.5)	

Distribution of the number of stones between groups A and B

Single stones were more common in both groups. The chi-square value was 0.57 with a p-value of 0.44, indicating that the difference in the distribution of single and multiple stones between the two groups is not statistically significant. The mean stone size was larger in Group A (10.07 ± 5.47 mm) than in Group B (8.96 ± 6.62 mm); however, the difference was not statistically significant (p = 0.41). The distribution of the number of stones between Group A and Group B is given in Table 4. 

**Table 4:** Distribution of the Number of Stones Between Group A and Group B. #Differences in the number of stones between Group A (standard-pressure pneumoperitoneum) and Group B (low-pressure pneumoperitoneum) were compared using the chi-square (χ²) test. Differences in the mean stone size between the two groups were compared using the unpaired t-test.

Number of stones	Group A, n (%)	Group B, n (%)	Test statistic^#^	p-value
Single	29 (72.5)	31 (77.5)	χ² value = 0.57	0.44
Multiple	11 (27.5)	9 (22.5)		
Mean size (mm)	10.07 ± 5.47	8.96 ± 6.62	t-test = 0.82	0.41

Intraoperative and postoperative details between groups A and B

The mean IAP in Group A was 11.51 ± 0.28 mmHg, compared to 9.50 ± 0.27 mmHg in Group B. The difference between the two groups was found to be statistically highly significant, as indicated by a p-value of 0.001, suggesting that Group A had significantly higher IAP compared to Group B. The mean operative time was 45 ± 9.2 minutes in Group B (low-pressure group) and 43 ± 8.5 minutes in Group A (standard-pressure group), with no statistically significant difference. Intraoperative complications were minimal and comparable between both groups.

Postoperative shoulder tip and abdominal pain scores were calculated at 6, 12, and 24 hours. There were no instances of shoulder tip pain between the two study groups at any time point. Additionally, for abdominal pain, no statistically significant difference was noted, indicating similar pain outcomes.

The mean duration of hospital stay was slightly higher in Group B (3.40 ± 0.84 days) as compared to Group A (3.18 ± 0.68 days), but this difference was not statistically significant (p = 0.19). The details are presented in Table 5.

**Table 5:** Comparison of Intraoperative Pressure, Postoperative Pain Scores, and Duration of Hospital Stay Between Group A and Group B Patients. #Differences in the mean values between Group A (standard-pressure pneumoperitoneum) and Group B (low-pressure pneumoperitoneum) were compared using the unpaired t-test. *Statistically significant difference between groups (p < 0.05). **Statistical comparison was not applicable (NA) between both groups as there was no incidence of shoulder tip pain at any time point. SD: standard deviation

Parameter	Group A, mean ± SD	Group B, mean ± SD	t-test^#^	p-value
Mean intra-abdominal pressure (mmHg)	11.51 ± 0.28	9.50 ± 0.27	32.3	0.001*
Operative time (minutes)	43 ± 8.5	45 ± 9.2	1.01	0.31
Postoperative shoulder tip pain scores				
At 6, 12, and 24 hours	0 ± 0	0 ± 0	NA**	NA**
Postoperative abdominal pain scores				
6 hours	5.75 ± 0.67	5.55 ± 0.81	1.19	0.23
12 hours	3.55 ± 0.55	3.57 ± 0.55	0.2	0.83
24 hours	1.82 ± 0.50	1.80 ± 0.52	0.21	0.83
Duration of hospital stay				
Mean (days)	3.18 ± 0.68	3.40 ± 0.84	1.31	0.19

Drain placement was not required in any patient. Although bleeding was observed in one patient (2.5%) in each Group A and Group B, there was no difficulty in achieving the critical view of safety (CVS) in either group, indicating similar intraoperative safety profiles. Port site complications, including infection, bleeding, or paralysis, were not reported in any patients across both groups. The details are presented in Table 6.

**Table 6:** Intraoperative and Postoperative Characteristics Between Group A and Group B Patients. CVS: critical view of safety

Operative and postoperative characteristics	Group A, n (%)	Group B, n (%)
Intraoperative complications		
Drain placement	0 (0)	0 (0)
Bleeding	1 (2.5)	1 (2.5)
Difficulty in achieving CVS	0 (0)	0 (0)
Postoperative complications		
Port-site infection/bleeding/ileus	0 (0)	0 (0)
Requirement of additional analgesics	0 (0)	0 (0)

## Discussion

In the present study, the demographic and clinical characteristics of study participants across Group A (standard-pressure) and Group B (low-pressure) demonstrated comparability, which is essential for ensuring the internal validity of the subsequent comparison of postoperative shoulder tip pain.

For shoulder tip pain, the results were unequivocal. Across all measured time points, the pain score was uniformly zero for every patient in both Group A (standard-pressure) and Group B (low-pressure). This complete absence of shoulder tip pain in our entire cohort is a striking finding, considering that the previous published literature, which assessed the efficacy of low- versus standard-pressure pneumoperitoneum, has consistently demonstrated that low-pressure has been associated with decreased postoperative shoulder tip pain.

Agarwal et al., in their prospective randomized study involving 100 patients, reported a lower incidence of shoulder tip pain in the low-pressure group (14% with a mean VAS score of 0.13) versus the standard-pressure group (38% with a mean VAS score of 0.92). They also reported lower analgesic requirement and hospital stays in the low-pressure group [11]. In their study consisting of 120 patients, Mahajan et al. also reported lower incidence of postoperative shoulder tip pain in the low-pressure groups; shoulder-tip pain was assessed using the Numeric Rating Scale (NRS). Approximately 30% of patients in the high-pressure group (mean NRS pain scale = 2.5) demonstrated postoperative shoulder tip pain, while only 8% of patients in the low-pressure group reported it (mean NRS pain scale = 1.2) [12]. Similar results were found by Sandhu et al., who also reported a lower incidence of shoulder tip pain in the low-pressure group [10]. Meena et al., in their study of 118 patients undergoing elective cholelithiasis, demonstrated similar results. The postoperative shoulder tip pain was recorded in 11.9% of patients in the low-pressure group (mean VAS score 0.14), versus 37.3% in the normal-pressure group (mean VAS score 0.97) [13]. For postoperative abdominal pain, at the 6-hour mark, both low-pressure and standard-pressure groups only reported moderate levels of pain; however, the difference was not statistically significant.

Several factors may have contributed to the complete absence of detectable shoulder-tip pain in our study, including the eligibility criteria, which excluded patients with complicated cholelithiasis. Additionally, the lack of a statistically significant difference in operative time in both study groups (<1 hour) may explain the similar pain profiles. However, while discussing operative time, it is important to note that Meena et al. [13] demonstrated an operative time even shorter than ours (26.10 minutes in the low-pressure group versus 25.47 minutes in the standard-pressure group), and they still demonstrated the presence of shoulder-tip pain in both pressure groups. This finding suggests that operative duration alone isn’t sufficient to explain the lack of shoulder-tip pain in the present study. 

Additionally, the standard analgesic protocol implemented in our study differed from the aforementioned studies. Agarwal et al. [11] and Meena et al. [13] used 100 mg tramadol on demand, and Mahajan et al. [12] used 75 mg diclofenac IV three times daily. Though we reserved tramadol as a rescue analgesic for patients with a VAS score of more than 7, no patient required it. These differences likely suggest that the standardized postoperative analgesic protocols may have masked the postoperative shoulder tip pain in our study. 

For surgical safety and intraoperative course, the results are similar between the two groups. Only one intraoperative complication was noted in each group (bleeding), and there were no reports of difficulty in achieving the CVS. This finding suggested that the lower-pressure pneumoperitoneum strategy was similar in comparison to the standard higher-pressure approach in terms of immediate intraoperative safety. The absence of an increase in complications in Group B suggests that simple procedures can be performed in a lower-pressure environment. 

Meena et al. evaluated intraoperative safety in low-pressure pneumoperitoneum as compared to standard-pressure pneumoperitoneum [13]. Complications such as bleeding and bile spillage were recorded, and there was no reported significant difference between the two groups. Similarly, Paria et al. conducted an observational study comparing low-pressure pneumoperitoneum with standard-pressure pneumoperitoneum [14]. The authors also reported similar findings: that low-pressure pneumoperitoneum did not compromise operative efficiency. These findings are consistent with the present study, where intraoperative complications were infrequent and comparable between the two pressure groups. The similarity in findings suggests that reducing pneumoperitoneum pressure does not adversely affect operative safety and supports the feasibility of low-pressure laparoscopic cholecystectomy without increasing the risk of intraoperative complications. However, within the limits of this study, low-pressure pneumoperitoneum does not prove to be beneficial in terms of a lower incidence of postoperative shoulder tip pain, as previously claimed by other studies. 

Limitations

The complete absence of the primary outcome in our cohort of 80 patients is a notable finding that prevented comparison between the two study groups. Several factors may have contributed to this finding, such as the postoperative analgesic protocol in our study, which may have suppressed shoulder tip pain in our study. Additionally, our cohort only included patients with uncomplicated cholelithiasis, limiting the applicability of the results to more complex clinical scenarios. Postoperative pain score was analyzed using the VAS, which is a subjective tool influenced by individual pain thresholds, psychological factors, and variations in analgesic response. Due to the complete absence of shoulder tip pain in our study, we could not establish the relationship between pressure difference in laparoscopic cholecystectomy and postoperative shoulder tip pain.

## Conclusions

The present study evaluated a cohort of 80 patients undergoing laparoscopic cholecystectomy for uncomplicated cholelithiasis. No postoperative shoulder tip pain was observed in either the low-pressure (8-10 mmHg) or standard-pressure (12-14 mmHg) study group at any of the assessed time points, thus preventing meaningful comparison between the two study groups. Additionally, reducing IAP did not confer any additional benefits in terms of postoperative pain relief or recovery in uncomplicated cases.

Therefore, in routine laparoscopic cholecystectomy, the choice of pneumoperitoneum pressure may be determined by the surgeon's preference, intraoperative visibility, and technical requirements. As multiple studies have claimed that low-pressure pneumoperitoneum affects postoperative shoulder tip pain, further large-scale, multicentric studies involving patients with difficult GB disease and longer follow-up periods are warranted to better define the role of low-pressure pneumoperitoneum in terms of shoulder tip pain.
